# Oncocytic Carcinoma with Liver Metastasis: Unusual Treatment Strategies and Clinical Insights—A Case Report and Review of the Literature

**DOI:** 10.1155/2024/3039762

**Published:** 2024-03-28

**Authors:** Mohammad Hourani, Selvaraj Giridharan, Fathi Azribi, Hidayath Ansari, Jawaher Ansari

**Affiliations:** ^1^Tawam Hospital, Al Ain, UAE; ^2^American Hospitals, Dubai, UAE; ^3^Cleveland Clinic, Abu Dhabi, UAE

## Abstract

We report a distinctive case of malignant oncocytic carcinoma originating in the pancreas, an organ rarely associated with such tumours. We discuss the diagnostic journey, highlighting the tumour's resemblance to renal cell carcinoma but without renal involvement. A significant aspect of this case is the successful and sustained response to combined immunotherapy and tyrosine kinase inhibitors, demonstrating a potential therapeutic pathway for similar rare cases. This study contributes to a deeper understanding of pancreatic oncocytic tumours and their management.

## 1. Introduction

Oncocytic tumours are a diverse group of neoplasms that can affect various organs. These tumours are characterized by oncocytes, cells exhibiting abnormal mitochondrial accumulation. The classification and prognosis of oncocytic tumours depend on the specific organ involved and the histological features the tumour displays. Oncocytic tumours can affect organs such as the adrenal glands, salivary glands, thyroid, kidney, and pancreas [1]. In the pancreas, oncocytic tumours can present as intraductal papillary mucinous neoplasms (IPMNs). These tumours are a unique type of pancreatic tumour defined by oncocytic cells [2]. Most oncocytic tumours are benign. However, this case highlights the complexity and rarity of malignant oncocytic carcinomas, especially those with metastatic potential. It underscores the importance of accurate diagnosis using histology and immunohistochemistry and explores the challenges and effectiveness of current treatment approaches, including surgical excision and innovative therapies like immunotherapy [3].

## 2. Case Report

**Figure 1:**
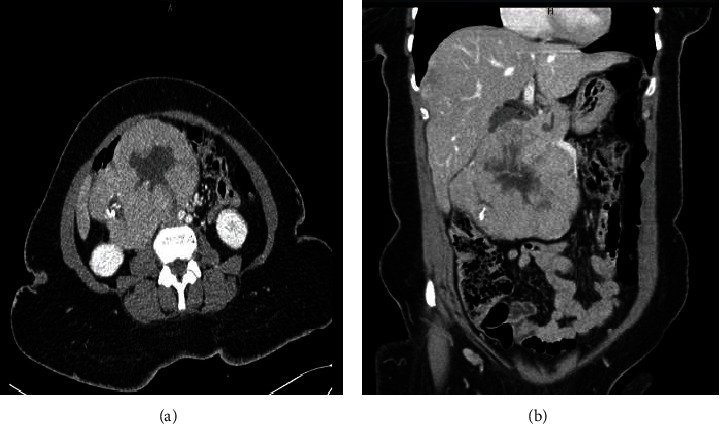
The postcontrast CT scan images in two orientations: (a) shows the axial view, while (b) depicts the coronal view.

A female patient, aged 56 years, with a medical background of well-managed diabetes and dyslipidaemia, was admitted to the hospital due to the presence of abdominal pain and a noticeable palpable mass in the abdominal area. Imaging revealed a 12.3 cm × 10.9 cm mass in the pancreas, with distinct features including intense enhancement and focal calcification, but no metastasis was initially detected (Figure 1). The patient's lab results showed elevated tumour markers CA 125 and CA 19-9 levels, while other lab values were within range. Based on the tumour board recommendation, the patient underwent a Whipple procedure.

The histopathology report of the resected specimen indicated the presence of an oncocytic neoplasm but with several characteristics commonly observed in renal cell carcinoma. These features included a distinct architectural pattern resembling renal cell carcinoma, abundant granular eosinophilic cytoplasm, and round nuclei with prominent nucleoli. Immunohistochemical staining was instrumental in characterizing the tumour, revealing positive staining for markers commonly associated with renal carcinomas, such as c-KIT and vimentin, while negative for CK7, typically expressed in pancreatic neoplasms. These findings suggest the possibility of a renal-type carcinoma originating in the pancreas, highlighting the tumour's unusual presentation and histological mimicry. Since there was no evidence of a primary renal lesion and the tumour was located in the pancreas, it was concluded that this was a rare instance of a primary pancreatic oncocytic carcinoma with renal-like histological features.

Postoperatively, the patient was under regular surveillance. After 42 months, liver metastases were identified. Biopsy of the liver lesion revealed an oncocytic neoplasm with immune profiling similar to the previous resection specimen. Molecular analysis of the metastatic oncocytic neoplasm indicated low microsatellite instability and an absence of actionable mutations, as determined by a comprehensive next-generation sequencing (NGS) panel. Additionally, the tumour demonstrated high PDL-1 expression (100%).

**Figure 2:**
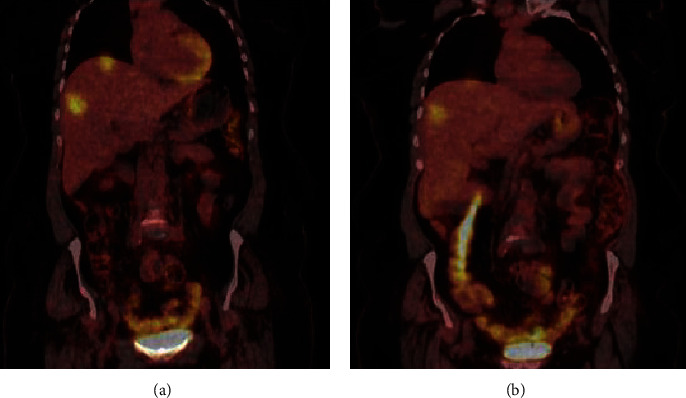
CT/PET images of the abdomen, highlighting the FDG uptake in liver metastases. (a) illustrates the condition before treatment, while (b) shows the changes following the completion of three treatment cycles.

The patient commenced a regimen of pembrolizumab, a PDL-1 inhibitor, and axitinib, a tyrosine kinase inhibitor. This treatment decision choice was based on several factors. Firstly, the high PDL-1 expression suggested a potential responsiveness to immunotherapy. This therapeutic avenue has been explored increasingly in the management of various malignancies, including renal cell carcinoma. Secondly, axitinib was chosen based on the tumour's immunohistochemical and histological characteristics, which predicted a favourable response to therapies commonly employed in renal carcinoma. This tailored that therapeutic approach underscores the significance of a detailed immunohistochemical and molecular characterization in guiding treatment decisions, particularly in cases where traditional diagnostic boundaries are blurred. This combination led to a partial response (Figure 2), though axitinib was later discontinued due to liver dysfunction. Lenvatinib was introduced alongside pembrolizumab, yielding radiological and clinical disease control with good tolerance 30 months into treatment.

## 3. Discussion

Oncocytes, also known as oxyphilic cells, are characterized by their large size, abundant granular cytoplasm, and deep eosinophilic staining, reflecting their high mitochondrial content [4]. These cells arise from epithelial origins and represent a distinct type of metaplastic transformation. Oncocytes are found in various organs under normal and pathological conditions, including the kidney, thyroid, liver, salivary glands, and parathyroid glands. Oncocytic change, often triggered by inflammatory or hyperplastic processes, leads to this granular cytoplasmic appearance. When oncocytic cells predominantly form a tumour, it is classified as an oncocytoma, generally benign, with a low propensity for metastasis [5]. However, there are rare malignant oncocytic carcinomas, which are aggressive and capable of metastasis. Research continues to explore oncocytomas, aiming to understand their behaviour and identify subsets with better prognoses.

In this case study, we discuss a rare pancreatic malignant oncocytic carcinoma resembling renal cell carcinoma but lacking chromophobe renal carcinoma traits. Initially treated with surgery, the patient later developed liver metastasis, effectively managed with pembrolizumab and TKI. This pancreatic oncocytoma case with renal carcinoma-like features is unprecedented in the medical literature. Renal oncocytic carcinomas, constituting 4.3% of renal tumours, are graded histologically from grade 1 (least aggressive) to grade 3 (most aggressive). Metastasis significantly worsens prognosis. Treatment typically involves surgery and sometimes radiotherapy, with rigorous follow-up due to the potential for malignancy, especially in pancreatic oncocytomas with endocrine features.

Low-grade renal oncocytomas (grade 1) are often considered premalignant, with no metastasis observed in such cases, as per Lieber et al. and Fairchild et al. Grade 2 tumours, rarer but more aggressive, show behaviours akin to renal carcinoma [6]. Metastasis in malignant renal oncocytomas can emerge years postsurgery [7]. Conservative management is standard for low-grade tumours, but surgery can be considered in bilateral cases [8]. First identified in 1942, renal oncocytoma incidence and awareness have risen, mainly affecting older adults. Links to genetic syndromes like TSC and BHD are documented, increasing the risk for these tumours.

The prevalence and incidence of oncocytomas, particularly in the context of renal neoplasms, are essential considerations in understanding these tumours' epidemiology and clinical significance. Guðbjartsson et al. report that the age-standardized incidence of oncocytomas was 0.3 per 100,000 per year for both men and women, with the incidence of oncocytomas being 5.5% of renal cell carcinomas (RCCs) diagnosed during the study period [9]. Additionally, Kim et al. indicate that oncocytoma has an overall incidence of 3% to 7% among all renal tumours and is the second most common benign tumour after angiomyolipoma [10]. Furthermore, Alanen et al. emphasize the increasing incidence of renal oncocytoma, urging for its recognition due to its better prognosis than renal cell carcinoma [11].

Initial findings indicate chromosomal alterations in renal oncocytomas, primarily in the nuclear genome, with possible mitochondrial changes [12]. These oncocytic changes might compensate for mitochondrial phosphorylate oxidation deficits. Mitochondrial myopathies, marked by oncocytic changes, stem from mitochondrial DNA alterations. Genetic anomalies in oncocytomas show various chromosomal changes, including 11q13 rearrangement affecting the CCND1 gene [13].

Initial imaging can reveal oncocytomas for renal masses, but further tests like biopsies are needed for definitive diagnosis. Despite radiologic ambiguity between benign and malignant lesions, certain features like central scars are indicative. Oncocytomas are often small, and a significant portion of small renal masses are benign, leading to the consideration of active surveillance [14]. Although biopsies provide insights into renal masses, accurately distinguishing between oncocytoma and chromophobe renal cell carcinoma (RCC) based solely on biopsy samples can be challenging. Due to the overlapping histological features of these tumours, a comprehensive evaluation that considers the entire clinical picture is often required. Genetic testing and advanced immunohistochemical analysis can offer additional clues in complex cases where conventional histopathology does not yield definitive results. While biopsies are generally reliable regarding diagnostic accuracy, the nuanced differentiation between oncocytoma and chromophobe RCC underscores the importance of a multifaceted diagnostic approach.

Treating oncocytoma poses challenges due to its rarity and the evolving understanding of effective treatments. Surgical removal is the primary approach for localized tumours, while systemic therapy may be necessary for extensive or inoperable metastases. Immunotherapy shows promise, with cases of metastatic renal oncocytoma responding to treatments like nivolumab. However, there is no established systemic therapy protocol, and chemotherapy and radiation have shown limited success. Surgery remains highly effective for most cases, but distinguishing benign oncocytomas from malignant forms requires careful histopathological examination.

## 4. Conclusion

Diagnosing malignant oncocytoma presents a complex challenge due to its morphological resemblance to benign oncocytomas. Accurate differentiation necessitates a multifaceted approach involving histological, immunohistochemical, electron microscopic, genetic sequencing, and clinical behaviour correlation. In cases of resectable lesions, surgical removal remains the preferred treatment modality. Our case, featuring metastatic liver lesions in a pancreatic oncocytoma, is unique and underscores the need for further research to understand this rare condition better, classify metastatic risks, and develop tailored treatments.
